# Supplementary material for: MyD88-dependent signaling drives toll-like receptor-induced trained immunity in macrophages
Source: Front Immunol. 2022 Nov 11;13:1044662. doi: 10.3389/fimmu.2022.1044662 (PMC9692127; doi:10.3389/fimmu.2022.1044662)
Supplement: Supplementary file 9 [file DataSheet_1.docx]

**Table 1. Primer sequences used for qRT-PCR gene expression analyses.**

| **Gene** | **Forward primer (5’→3’)** | **Reverse primer (5’→3’)** |
| --- | --- | --- |
| *MARCO* | CCTCCAGGGACTTACGGGT | CCAGTGAGACCTATGTCACCT |
| *PECAM1* | ACGCTGGTGCTCTATGCAAG | TCAGTTGCTGCCCATTCATCA |
| *Ptprm* | CATGCTGGTCAACACTTCTGG | GCAGCGTTACTCTTGCTGGATA |

**Supplemental Figure 1. F4/80 immunohistochemical staining of kidney samples harvested 1-day after *S. aureus* infection in macrophage-depleted mice compared to controls.** (**A**) Representative low magnification (10X) micrographs were captured (scale bar represents 100 μm) and (**B**) threshold processing was conducted for quantification using ImageJ in clodronate liposome (CL)-treated or PBS liposome (PL)-treated mice administered TLR agonists CpG or Poly I:C (20 μg) alongside vehicle controls (0.2 mL *i.v.*).

**Supplemental Figure 2. Pearson correlation analysis demonstrates high consistency among biological replicates.** Pearson correlation analysis on WT, MyD88 KO (MKO), or TRIF KO (TKO) RNA samples from unstimulated (US) or 3dp MPLA (M) bone marrow derived macrophage conditions demonstrates the degree of correlation (R^2^) among biological replicates (n = 3/group). Darker shading and higher R^2^ value indicate stronger linear correlation.

**Supplemental Figure 3. Downregulated enrichment pathways in MPLA-treated MyD88 KO (MKO) or TRIF KO (TKO) compared to MPLA-treated wild type (WT) macrophages.** The 5 most downregulated enrichment pathways of each gene ontology (GO) class (biological process, cellular component, and molecular function) in MPLA-treated MKO cells compared to MPLA-treated WT cells were identified. These identified pathways were then assessed for MPLA-treated TKO cells compared to MPLA-treated WT macrophages, and the degree of significance is displayed (-log padj).

**Supplemental Figure 4. TLR2/MyD88-activating agonist Pam3CSK triggers macrophage metabolic reprogramming.** Bone marrow-derived macrophages (BMDMs) were treated with Pam3CSK (P3C; 1 µg/mL) for 24 hours and allowed to rest for 3 days compared to unstimulated (US) controls. (**A**) Intracellular ATP was measured. (**B**) Mitochondrial content was assessed by MitoTracker Green staining and (**C**) mitochondrial membrane potential was assessed by TMRM staining and measured by flow cytometry with mean fluorescent intensity (MFI) shown. (**D**) BMDMs from wild type (WT), MyD88 KO, and TRIF KO mice were treated with P3C as described and compared to US controls. Peak glycolysis was measured as indicated by Extracellular Acidification Rate (ECAR), and (**E**) peak mitochondrial respiration was measured by oxygen consumption rate (OCR). Data are shown as mean ± SEM, n = 3-5/group, * *p* < 0.05, *** *p* < 0.001, **** *p* < 0.0001 by ANOVA followed by Dunnett’s post-hoc multiple comparison test.

**Supplemental Figure 5. Cluster analysis of metabolism-related genes demonstrates distance of MPLA-treated MyD88 KO macrophages from MPLA-treated wild type and TRIF KO macrophages.** WT, MKO, and TKO bone marrow-derived macrophages (BMDMs) were treated with MPLA for 24h and rested for 3 days (_M) or left unstimulated as controls (_US) and RNA was harvested for RNAseq analysis. (**A**) Normalized expression of metabolism-related genes (list provided in Figure 5A) in individual samples (represented by columns, n=3/group) represented as a heatmap. Complete-linkage hierarchical clustering was performed using correlation-based distance. (**B**) Hierarchical clustering tree (using parameters described in panel A) for genes with maximum expression level at the top 75%. (**C**) Pearson’s Correlation matrix demonstrating stronger positive correlation (r value closer to 1) in red and weaker correlation in green.

**Supplemental Figure 6. Cluster analysis of antimicrobial function-related genes demonstrates distance of MPLA-treated MyD88 KO macrophages from MPLA-treated wild type and TRIF KO macrophages.** WT, MKO, and TKO bone marrow-derived macrophages (BMDMs) were treated with MPLA for 24h and rested for 3 days (_M) or left unstimulated as controls (_US) and RNA was harvested for RNAseq analysis. (**A**) Normalized expression of antimicrobial function-related genes (list provided in Figure 6C) in individual samples (represented by columns, n=3/group) represented as a heatmap. Complete-linkage hierarchical clustering was performed using correlation-based distance. (**B**) Hierarchical clustering tree (using parameters described in panel A) for genes with maximum expression level at the top 75%. (**C**) Pearson’s Correlation matrix demonstrating stronger positive correlation (r value closer to 1) in red and weaker correlation in green.

**Supplemental Figure 7. TLR2/MyD88-stimulating agonist Pam3CSK augments antimicrobial capacity of BMDMs.** Bone marrow-derived macrophages (BMDMs) from wild type (WT), MyD88 KO, and TRIF KO mice were treated with Pam3CSK (P3C; 1 µg/mL) for 24 hours and allowed to rest for 3 days compared to unstimulated (US) controls. (**A**) Phagocytic capacity and (**B**) reactive oxygen species (ROS) production capacity were measured.. Data are shown as mean ± SEM, n = 2-7/group, * *p* < 0.05, *** *p* < 0.001 by Student’s T-test.

**Supplemental Figure 8. Splenomegaly was observed up to 1-week after CpG treatment and F4/80+ splenic macrophages were immunomagnetically isolated for downstream analysis.** (**A**) Spleen weight (mg) was measured 1d, 3d, 1-week, and 2-weeks after CpG treatment (20µg *i.v.*; n=4-16/group). (**B**) Percent F4/80^+^ macrophages in splenocyte cell suspension before (left) and after (right) magnetic isolation as assessed by flow cytometry. **** *p* < 0.0001 by ANOVA followed by Dunnett’s post-hoc multiple comparison test.
